# The miRNA Plasma Signature in Response to Acute Aerobic Exercise and Endurance Training

**DOI:** 10.1371/journal.pone.0087308

**Published:** 2014-02-19

**Authors:** Søren Nielsen, Thorbjörn Åkerström, Anders Rinnov, Christina Yfanti, Camilla Scheele, Bente K. Pedersen, Matthew J. Laye

**Affiliations:** 1 The Centre of Inflammation and Metabolism, Department of Infectious Diseases and CMRC, Rigshospitalet, Faculty of Health Sciences, University of Copenhagen, Copenhagen, Denmark; 2 The Buck Institute for Research on Aging, Novato, California, United States of America; 3 August Krogh Centre, Faculty of Science, University of Copenhagen, Copenhagen, Denmark; GDC, Germany

## Abstract

MiRNAs are potent intracellular posttranscriptional regulators and are also selectively secreted into the circulation in a cell-specific fashion. Global changes in miRNA expression in skeletal muscle in response to endurance exercise training have been reported. Therefore, our aim was to establish the miRNA signature in human plasma in response to acute exercise and chronic endurance training by utilizing a novel methodological approach. RNA was isolated from human plasma collected from young healthy men before and after an acute endurance exercise bout and following 12 weeks of endurance training. Global miRNA (742 miRNAs) measurements were performed as a screening to identify detectable miRNAs in plasma. Using customized qPCR panels we quantified the expression levels of miRNAs detected in the screening procedure (188 miRNAs). We demonstrate a dynamic regulation of circulating miRNA (ci-miRNA) levels following 0 hour (miR-106a, miR-221, miR-30b, miR-151-5p, let-7i, miR-146, miR-652 and miR-151-3p), 1 hour (miR-338-3p, miR-330-3p, miR-223, miR-139-5p and miR-143) and 3 hours (miR-1) after an acute exercise bout (P<0.00032). Where ci-miRNAs were all downregulated immediately after an acute exercise bout (0 hour) the 1 and 3 hour post exercise timepoints were followed by upregulations. In response to chronic training, we identified seven ci-miRNAs with decreased levels in plasma (miR-342-3p, let-7d, miR-766, miR-25, miR-148a, miR-185 and miR-21) and two miRNAs that were present at higher levels after the training period (miR-103 and miR-107) (P<0.00032). In conclusion, acute exercise and chronic endurance training, likely through specific mechanisms unique to each stimulus, robustly modify the miRNA signature of human plasma.

## Introduction

MiRNAs are 20-24 nucleotides non-coding RNAs that regulate protein abundance primarily by binding to the 3′UTR of coding mRNAs [1]. A single miRNA can regulate the expression of hundreds of mRNAs and proteins [2], [3] and are therefore significant player in regulating many aspects of biology [4]–[6]. Several miRNAs have been shown to be modulators of biological pathways in skeletal and cardiac muscle hypertrophy and metabolism [7]–[9]. Aerobic and resistance exercise interventions alter the global transcriptional miRNA pattern of these tissues [10]–[13]. These changes in miRNA expression provide numerous novel explanations for the effects of training on metabolism. For instance, an acute aerobic exercise intervention down regulates miR-23a expression in skeletal muscle in both humans and mice [12], [14]. MiR-23a is verified as a direct target for PGC-1α [15], a regulator of mitochondrial biogenesis [16]. The exercise induced alteration in miRNA expression is therefore a likely muscular adaptation mechanism in response to endurance training.

Recently it was demonstrated that mature miRNAs are detectable in the circulatory system [17] and they are therefore studied as biomarkers for different diseases [18]. Packed in exosome vesicles, miRNAs are released to the circulation by most cell types, including skeletal muscle [19]. Characterisation of the exosome membranes has revealed presence of transmembrane proteins, which makes it possible for exosomes to bind to specific recipient target cells and deliver the vesicle content [20]. Indeed, several studies have demonstrated an exosome mediated cell-cell functional miRNA delivery [21]–[23].

A few studies have shown that increased physical activity is associated with altered levels of circulating miRNAs (ci-miRNAs). Baggish and co –workers demonstrated altered expression of selected ci-miRNAs in response to both acute and chronic exercise interventions [24]. The eight selected ci- miRNAs had distinct expression profiles in response to these interventions. Another study has demonstrated that low maximal aerobic oxygen consumption is associated with high expression levels of three different ci-miRNAs [25]. In response to exercise skeletal muscle releases myokines, but whether exosomes are also secreted is unknown [26]. However, in vitro studies have revealed that myotubes has the capability of releasing exosomes [27] suggesting that skeletal muscle contributes to altered ci-miRNA levels.

Importantly, endurance exercise and training affects the transcriptional machinery in many other organs than skeletal muscle [28]–[30]. The human genome encodes 2578 mature miRNAs [31] and each tissue has its own specific miRNA expression fingerprint [32]. Those tissues can potentially secrete exosomes to the circulation and thereby contribute to an altered ci-miRNA signature. To date, no global screening of ci-miRNAs in response to endurance exercise and training has been reported.

Interestingly, new studies describing the technical challenges when measuring miRNAs in plasma and serum have drawn a lot of attention to the field of circulating miRNAs [33], [34]. Several important pre-analytical considerations are crucial for achieving exact miRNA quantifications. Firstly, the majority of ci-miRNAs are present inside red blood cells [35] and minor degrees of hemolysis can therefore contaminate the plasma ci-miRNA expression pattern. Thus, quality control strategies are of great importance to avoid inclusion of hemolyzed plasma samples in the data set. Secondly, accurate measurements are further challenged by low RNA yields and finding reliable “endogenous” controls for qPCR normalisation. Taken together, measuring miRNAs in plasma is a method with several pitfalls that needs to be considered to avoid false positive or negative results.

Using a global screening approach, the overall aim of this study was to investigate the ci-miRNA plasma signature of humans in response to acute endurance exercise and training in plasma samples from healthy young men. An additional aim was to establish a reliable methodology for relative quantification of miRNAs in human plasma.

## Methods

### Ethical Approval

The study was approved by the local Ethical Committee of Copenhagen and Frederiksberg (KF 01 289434) and was performed in accordance with the Declaration of Helsinki. The purpose of the study and its possible risks and discomforts were explained to the participants before their written consent was obtained.

### Subjects

**Figure 1:**
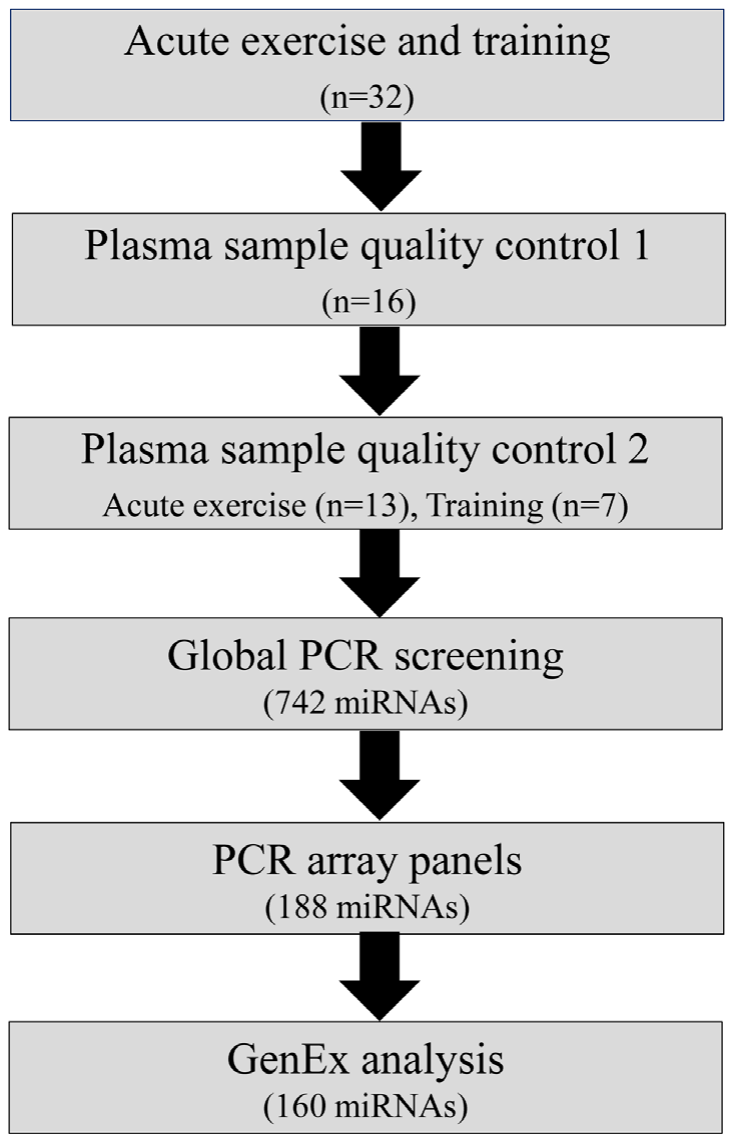
Study workflow. Thirty two healthy young men were included in the study, Where Plasma samples from 16 subject passed the first quality control for hemolysis, plasma samples from thirteen subjects passed the other. Seven plasma samples 12 weeks endurance training intervention passed the second control. A global miRNA PCR screening was performed to identify ci-miRNA expression levels (742 miNRAs). Based in on the global screening a customised PCR arrays including 188 miRNA primers were designed. The PCR data was analysed using the GenEx analysis tool where 160 miRNAs were detectable.

Thirty two healthy, trained men were included in the study. Quality control (described below) analysis of plasma samples indicated that thirteen passed for the acute exercise analysis and seven for the endurance training analysis (Figure 1). The Subjects included was part of a large training study investigating the effect of glucose and antioxidant ingestion on training adaptation [36], [37] Subject characteristics are listed in Table 1. Before inclusion in the study, a medical examination with blood test screening, a test for maximal power output (*P*_max_), and an oral glucose tolerance test were performed. Exclusion criteria included regular physical exercise less than 2 and more than 4 times per week, BMI>30 kg m^−2^, smoking and impaired glucose tolerance [36].

**Table 1:** Subject characteristics.

	Acute exercise	Endurance training	
Subject characteristics	Pre training(n = 13)	Pre(n = 7)	Post-(n = 7)
Age	28±8	28±5	n/a
Height	182.3±8.3	185.1±8.1	n/a
Weight (kg)	79.9±8.4	84.63	83.2
**Fitness**			
VO2 max (l/min/kg)	52.6±4.8	52.1±5.9	62.6±4.2**
Pmax (Watt)	346±45	372±38	440±38*

Data are mean ± SD. *P<0.05, **P<0.001.

### Exercise Trial and Training Programme

The endurance exercise trial was performed as previously described [36]. Briefly, before and after the training period the subjects performed a 60 min cycle ergometer exercise bout at 65% of *P*_max_ (Monark 839E, Monark Ltd, Varberg, Sweden). On the experimental day, subjects arrived, in a fasted state, at the lab between 07.30 and 08.00 h. After exercise the subjects rested on a hospital bed for 180 min. The subjects trained, under supervision, on a cycle ergometer (Monark 839E) with frequency of 5 times per week for 12 weeks as previously reported [37]. The participants were instructed to maintain their habitual diets. Furthermore the subjects were instructed not to eat 2 hours prior to the daily exercise bouts.

### Plasma Samples and Quality Control

Blood samples were drawn at rest immediately before exercise, immediately after a 1 h acute exercise bout, 1 h post exercise, 3 h post exercise and post 12 weeks of endurance training at rest. The blood samples were drawn from an antecubital vein into EDTA-containing glass tubes (Vacutte, Greiner bio-one), and they were immediately centrifuged at 3,500 *g* for 15 min at 4°C. Plasma samples were then stored at −80°C until analysed. 1.5 µl of plasma was analysed spectrophotometrically (NanoDrop 1000, Thermo Scientific) to determine levels of free haemoglobin in the samples [33]. Absorbance levels above 0.2 at 414 nm were indicative of free haemoglobin and thereby higher degree of haemolysis and such samples were excluded from further analysis.

### RNA Isolation from Human Plasma

To remove cell debris, 500 µl of plasma was transferred to a 1.5 ml tube and centrifuged (5415 R, Eppendorf) at 1000 g for 5 minutes at 4°C. Next, 200 µl of the supernatant was transferred to a new 1.5 ml tube and mixed with 750 µl lysis mixture containing Qiazol lysis reagent (Qiagen, Maryland, US) with 1.25 µl 0.8 µg/µl MS2 RNA to improve RNA yield and 1 µl Spike-in mix (UniSp2, UniSp4, UniSp5 RNA) (Exiqon, Vedbaek, Denmark) to control for differences in isolation efficiency. Subsequently, 200 µl chloform was added to the samples and followed by vortex for 10 seconds. After 2 minutes incubation at RT the samples were centrifuged at 12,000 g for 15 minutes at 4°C. The upper aqueous phase was then transferred to a 2 ml tube. 1.5 mL 99.9% ethanol (Sigma-Aldrich, St. Louis, US) was added to the samples which followed by 7 seconds of vortex. Samples were transferred to RNeasy mini spin columns (Qiagen, Maryland, US) that were centrifuged at 13,000 g. After washing with RWT buffer and RPE buffer (Qiagen, Maryland, US) the RNA pellet was dried for approximately 1 minute. Lastly, 50 µl water was added to dissolve the RNA. Dissolved RNA was collected in 1.5 ml tubes after centrifugation at 13,000 g. Samples were stored at −80°C.

### cDNA Synthesis of Human Plasma miRNA

RNA concentration in isolations from plasma cannot be determined accurately due to RNA yield being below detection range when measured on a spectrophotometer. Thus 200 µl of starting material (plasma) was used as a constant input amount in the cDNA synthesis. Isolated plasma RNA was diluted 50× and an additional synthetic spike-in RNA (UniSp6 RNA) (Exiqon, Vedbaek, Denmark) was added to control for differences in cDNA efficiency. The cDNA reaction enzymes (Exiqon, Vedbaek, Denmark) were then mixed with diluted cDNA according to the manufacturer’s protocol.

### Quantitative Real-time PCR Quality Control

In addition to the sample quality control performed spectrophotometrically (NanoDrop 1000, Thermo Scientific) an additional sample control was performed by quantitative real-time PCR (qPCR). The RNA isolation efficiency was controlled by using PCR primers targeting the synthetic spike in RNAs added in the lysis reagent during RNA isolation. Additionally, cDNA synthesis efficiency was controlled for by using a primer targeting the spike in (UniSp6 RNA) added in the cDNA synthesis step. Samples that diverged more that 25% from the average of calculated absolute values were excluded from the study (workflow figure 1). In addition, a qPCR analysis for erythrocyte enriched miR-451 was performed. Furthermore, the expression of previously described stable plasma ci-miRNAs, miR-103, miR-423-5p and miR-191 was performed. PCR plates included primers for all eight control targets (spike-in RNAs and endogenous RNAs) on Exiqons Pick-&-Mix customised 96 well PCR panels. CDNA samples were mixed with SYBR green and loaded to the custom panels and quantitative qPCR was performed using the ViiA7 Sequence Detection system (Applied Biosystems, Foster City, CA, USA) according to the manufacturer’s protocol.

### Global miRNA Screening – Selection Procedure

To identify which miRNAs that are expressed in the circulation we performed a global miRNA screening using Exiqons miRNome panel V.2 that includes primers for 752 miRNAs. cDNA samples for each timepoint were pooled and loaded to the panels mixed with SYBR green and ROX in proportions recommended by the manufacturer. From the miRNome PCR panel, 188 miRNAs were evaluated to be expressed when Ct- values were lower than 33.5.

### MiRNA Quantification with qPCR Panels

Plasma PCR panels including the 188 selected miRNAs were designed using the Pick-&-Mix microRNA system (Exiqon, Vedbaek, Denmark). Each cDNA sample was mixed with SYBR green and ROX dye in proportions recommended by the manufacturer and loaded to the panels. The ViiA7 Sequence Detection system was programmed to perform a melting curve analysis to check for primer specificity. Data was exported from the ViiA7 Sequence Detection system and imported in the multi PCR data analysis system GenEx Enterprise (Exiqon, Vedbaek, Denmark). To control for variation between samples an endogenous ci-miRNA control was selected using the Genorm and normfinder algorithms included in the GenEx Enterprise software, which determine the miRNA that varies least between all samples and assumed to be the most suitable as an endogenous control. From the 188 miRNAs detected in the first global screening using pooled cDNA, 160 miRNAs were detectable when analysing each individual sample.

### Statistics

Subject characteristics, performance data and qPCR Ct-values are presented as mean ± SD as stated in the tables. Data in the figures are presented as mean ± SEM. To minimize false positives results the 0.05 significance level for multiple testing was corrected using the dunn-bonferroni correction method. Samples with p-values below 0.00032 were therefore considered as significant altered. An additional borderline significance level was set to 0.001 to ensure that all likely differentially expressed miRNAs were detected. A significance levels at 0.001 means that 0.16 of the 160 ci-miRNAS will be differentially expressed by chance. Quantification of PCR data was performed using the delta-delta ct-method in the Genex enterprise PCR calculation tool (Exiqon, Vedbaek, Denmark). MiRNA Expression levels were tested for normal distribution and equal variance and thereafter for statistical significance using paired t-tests.

## Results

### Blood Plasma Quality and Physiological Response to 12 Weeks of Endurance Training

Plasma samples from 32 healthy young men were analysed for absorbance peaks at 414 nm spectrophotometrically to determine degree of hemolysis (workflow in figure 1). Plasma samples from 16 participants were estimated to have a purity that passed the critical level based on an absorbance threshold below 0.2 at 414 nm, as described by Kirschner et al. [33]. The plasma samples from the sixteen participants went through a second quality control step where we spiked synthetic RNAs into each sample during the RNA isolation and cDNA synthesis procedure, to ensure equal isolation and cDNA efficiencies respectively. We noted that the expression levels of spike-in RNA added during the RNA isolation and cDNA synthesis step did not vary between samples when quantifying with qPCR, suggesting equivalent RNA isolation and cDNA synthesis (data not shown). Next, as an additional control for hemolysis we measured expression of miR-451 which is highly enriched in erythrocytes [38]. Plasma from 13 of the 16 subjects with equal miR-451 expression passed the second quality control step. However, 6 of the 13 post training plasma samples revealed a likely hemolysis contamination and were therefore excluded before running the arrays. The physiological data of included participants at basal and in response to 12 weeks of training is given in Table 1.

### Ci-miRNAs are Down-regulated Immediately after an Acute Exercise Bout

To test whether levels of ci-miRNAs was regulated by acute endurance exercise; subjects underwent 60 min of cycling at 65% of Peak Watt. Blood was drawn from an antecubital vein before the exercise bout, immediately after, 1 h post exercise and 3 h after exercise. Differentially expressed ci-miRNAs that passed the Bonferroni cut-off (P<0.00032) are shown in figure 1. Eight ci-miRNAs (miR-106a, miR-221, miR-30b, miR-151-5p, let-7i, miR-146a, miR-652, miR-151-3p) were robustly down-regulated immediately after the exercise bout, indicating a blood miRNA clearance possibly as a result of uptake of specific miRNAs from surrounding tissues or urea clearance. Thus, specific ci-miRNAs seems to rapidly adjust to the increased physical activity level.

### Ci-miRNAs are Up-regulated 1 h–3 h after Acute Endurance Exercise

**Figure 2:**
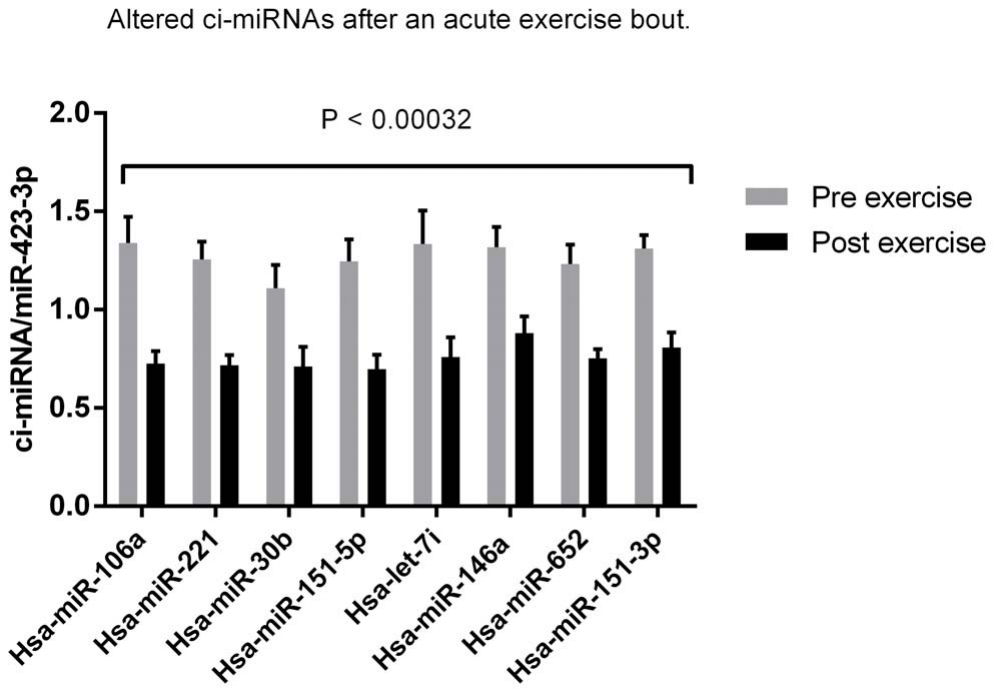
Alterations in ci-miRNAs in plasma immediately after an acute exercise bout. Eight ci-miRNAs were down regulated after a single 60 min aerobic exercise bout at 65% Pmax. Ci-miRNAs below the Dunn-bonferroni corrected p-value P<0.00032 was considered as significant altered.

**Figure 3:**
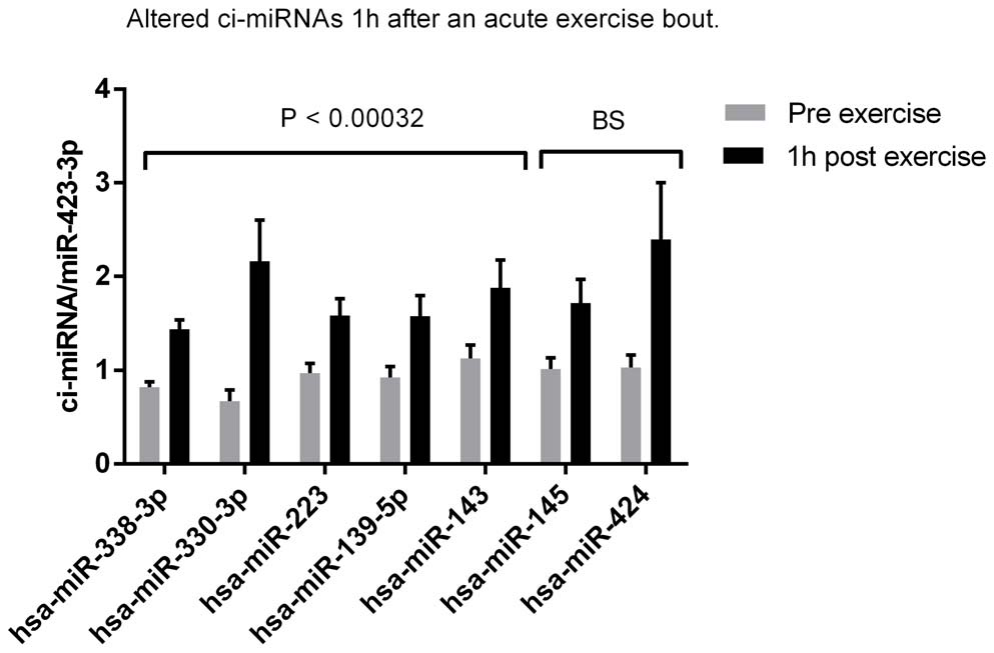
Alterations in resting ci-miRNAs in plasma one hour after an acute exercise bout. Five ci-miRNAs were up regulated 1 hour after a 60 min aerobic exercise bout at 65% Pmax when Dunn-bonferroni correction of the significance level 0.05 was performed (P<0.00032). Additional two miRNAs were borderline significant (BS) up regulated at significance level 0.001.

In contrast to the observation immediately after endurance exercise, several differentially expressed ci-miRNAs were up-regulated 1 h and 3 h post exercise (Figure 2 and Figure 3). One hour after the acute exercise bout 5 miRNAs (miR-338-3p, miR-330-3p, miR-223, miR-139-5p, miR-143) were upregulated and after additional 2 hours 1 miRNA was increased (miR-1). In addition to the ci-miRNAs that passed the bonferroni cut-off (P<0.00032), 4 borderline significant ci-miRNAs (miR-145, miR-424, miR-133a, miR-133b) (P<0.001), were also identified (Figure 2 and Figure 3). Among those are the muscle specific miRNAs (mir-1, miR-133a and miR-133b) that are up regulated 3 h after the exercise bout. In line with that observation, we and others have previously shown that miR-1 and miR-133a expression is induced in muscle following acute aerobic exercise [14], [37]. In addition, the clustered miRNAs miR-143 and miR-145 were up-regulated 1 h after exercise (figure 3). Both miRNAs are enriched in liver [39], suggesting that other tissues than muscle may secrete miRNAs in response to endurance exercise. MiR-424 that control vascular smooth muscle phenotype [40] was up-regulated 1 h and 3 h after the exercise bout. The observation indicates that organs affected by endurance exercise secrete miRNA to the circulation as a response to the stimuli. Summary of all ci-miRNAs that were differentially expressed in the current study, their possible tissue origin and expression levels are listed in Table 2.

**Table 2:** CT values are presented as mean ± SD.

Pre exercise -post exercise					
miRNA	Alterations Δ%	CT	P value	Possible tissue origin	Reference
miR-106	−84%	27.5±1.6	P<0.00032	Bone marrow	Garzon 2006
miR-221	−75%	29.5±1.5	P<0.00032	Angiogenic regulator	Poliseno 2006
miR-30b	−56%	30.2±1.8	P<0.00032	Endothelial cells	Bridge et al. 2012
miR-151-5p	−78%	29.9±1.7	P<0.00032	Liver	Luedde et al. 2010
let-7i	−75%	30.8±1.5	P<0.00032	Most tissues	–
miR-146a	−49%	30.3±1.6	P<0.00032	Immune system	Terrazas et al. 2013
miR-652	−63%	30.5±1.5	P<0.00032	No specific tissue	–
miR-151-3p	−62%	30.5±1.6	P<0.00032	No specific tissue	–
**Pre exercise** −**1 h post exercise**					
miR-338-3p	74%	31.5±1.4	P<0.00032	beta cells	Jacovetti et 2013
miR-330-3p	222%	35.6±2.5	P<0.00032	No specific tissue	–
miR-223	63%	24.5±2.0	P<0.00032	Cardicac	Lu. Et al 2009,
miR-139-5p	70%	32.1±1.4	P<0.00032	No specific tissue	–
miR-143	66%	31.4±1.4	P<0.00032	Liver	Jordan et al 2011
miR-145	68%	31.2±1.4	P<0.001	Liver	Jordan et al 2011
miR-424	132%	28.9±1.4	P<0.001	Smooth muscle cells	Merlet et al 2013.
**Pre exercise** −**3 h post exercise**					
miR-1	429%	32.9±1.9	P<0.00032	Cardiac,Skeletal muscle	Nielsen et al. 2010, Olson et al. 2007
miR-424	63%	28.9±1.4	P<0.001		Merlet et al 2013.
miR-133a	252%	34.8±1.6	P<0.001	Cardiac, Skeletal muscle	Nielsen et al. 2010, Olson et al. 2007
miR-133b	454%	32.9±1.7	P<0.001	Skeletal Muscle	Nielsen et al. 2010, Olson et al. 2007
**Pre training -post trainig**					
miR-342-3p	−207%	30.8±1.3	P<0.00032	No specific tissue	–
let-7d	−194%	33.9±2.1	P<0.00032	No specific tissue	–
miR-103	124%	28.7±1.9	P<0.00032	Liver, adipose	Moncine et al. 2010
miR-107	127%	29.9±2.1	P<0.00032	Liver, adipose	Moncine et al. 2011
miR-766	−69%	32.6±2.0	P<0.00032	No specific tissue	–
miR-25	−124%	30.5±1.2	P<0.00032	Cardiac	Cao et al. 2012
miR-148a	−225%	30.3±1.6	P<0.00032	No specific tissue	–
miR-185	−183%	28.1±1.1	P<0.00032	Brain	Zhang 2011
miR-21	−184%	26.5±1.5	P<0.00032	Immune system	Xu. et al 2013
miR-148b	−194%	29.8±1.5	P<0.001	No specific tissue	–
miR-133a	−228%	34.8±1.6	P<0.001	Cardiac,Skeletal muscle	Nielsen et al. 2010, Olson et al. 2007
miR-92a	−214%	26.8±1.3	P<0.001	Muscle	Kellet et al. 2011
miR-29b	−70%	32.9±1.8	P<0.001	Muscle	Kellet et al. 2011

CT values are presented as mean ± SD.

### Basal ci-miRNA Levels are Altered after 12 Weeks Endurance Training

**Figure 4:**
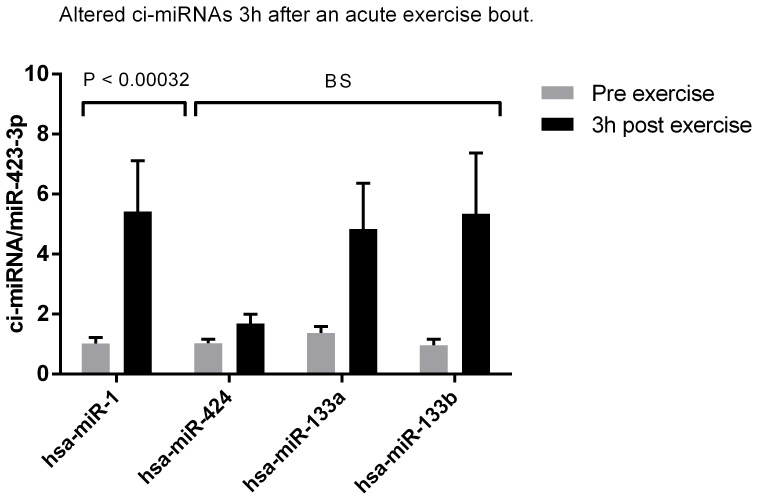
Alterations in ci-miRNAs in plasma three hours after an acute exercise bout. One ci-miRNA was up regulated 3 hour after a 60 min aerobic exercise bout at 65% Pmax when Dunn-bonferroni correction of the significance level 0.05 was performed (P<0.00032). Additional three miRNAs were borderline significant (BS) up regulated at significance level 0.001.

**Figure 5:**
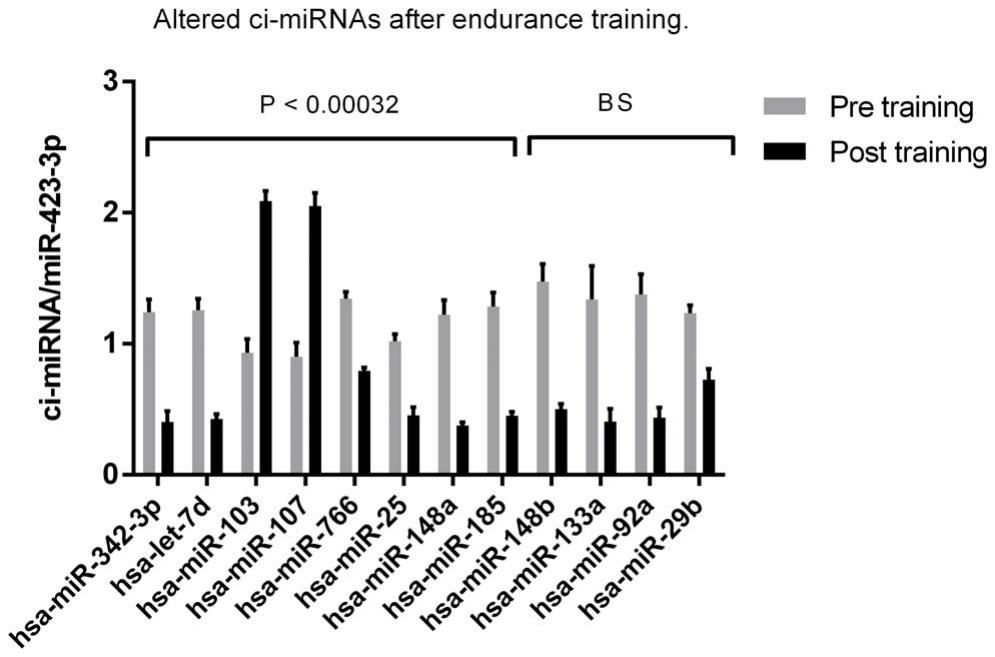
Alterations in ci-miRNAs in plasma in response to 12 weeks of endurance training. Two ci-miRNAs were up regulated and seven were down regulated in response to 12 weeks endurance training when Dunn-bonferroni correction of the significance level 0.05 was performed (P<0.00032). Additional four miRNAs were borderline significant (BS) down regulated at significance level 0.001.

We measured resting ci-miRNAs in plasma before and 3–5 days after 12 weeks endurance training to identify the possible chronic plasma alterations. In addition to the 9 ci-miRNAs that passed the bonferroni cut-off (P<0.00032), we included 4 border line significant ci-miRNAs (P<0.001) (Figure 3, 4 and 5). Seven ci-miRNAs were significantly decreased (miR-342-3p, let-7d, miR-766, miR-25, miR-148a, miR-185, miR-21), while two ci-miRNAs were significantly increased after the training period (miR-103, miR-107) (P<0.00032). In addition, four borderline significantly ci-miRNAs (miR-148b, miR-133a, miR-92a, miR-29) were found (Figure 5) (P<0.001).

## Discussion

In the current study, we employed multiple quality control steps to tightly control a ci-miRNA screen aimed at identifying global alterations in ci-miRNAs following acute exercise and endurance training. Immediately after an acute exercise bout the levels of eight ci-miRNAs were robustly decreased. In contrast, one and three hours post exercise more ci-miRNAs increased than decreased. However, in plasma samples collected at rest before and after endurance training a range of alterations in both directions was observed.

The general decrease in ci-miRNAs observed immediately after acute exercise was an intriguing observation. We carefully went through the raw data set to examine the expression levels of the reference ci-miRNA miR-423-3p. No systematic variation between time points was observed (P>0.05). Therefore, the data suggests exercise induces clearance of specific ci-miRNAs from the circulation. Interestingly, a study by Vickers et al. revealed that in addition to the traditional extracellular transport of miRNAs by exosomes, an additional transport mechanism by lipoproteins exists [41]. Acute exercise and endurance training reduce the levels of circulating Low-density lipoproteins (LDL) [42]. Therefore, it is indeed possible that lipid proteins associated with ci-miRNAs are cleared from the circulation in response acute exercise. In support, a human training study by Aoi and co-workers showed that the muscle enriched miR-486 was decreased in response to acute and chronic aerobic exercise [43].

Importantly, the acute exercise induced decreases in circulating miR-221 and miR-146a is in contrast to the finding by a recent published study by Baggish et al. [24]. In that study, the basal expression of both these ci-miRNAs was robustly elevated in response to acute exercise and at basal after 10 weeks of endurance rowing training. In the current study neither miR-221 nor miR-146 were differentially expressed after 12 weeks of endurance training. In addition, the endurance training and acute exercise induced response in terms of miR-21 expression was in contradiction to the current study. Where acute exercise had no effect on miR-21 expression, endurance training markedly decreased the expression. However, our observed decrease is in line with the finding by Bye et al. who demonstrated an association between low maximal oxygen consumption and high levels of circulating miR-21 expression [25]. A number of reasons could explain the discrepancies between the two data sets. The acute exercise bouts differed between the two studies. Where the subjects in the current study exercised for 1 h at 65% of Pmax, Baggish et al. utilised a cycling incremental VO2 max test as an acute exercise intervention. Furthermore, the post-processing of samples differed dramatically. We utilised a stable expressed ci-miRNA to account for the biological variation between samples, instead of the synthetic spike in approach used by Baggish et al. Both normalisation strategies are widely used in the literature but will most likely contribute to differences between studies. In addition to the biological endogenous control, we included two additional normalisation strategies where synthetic spike RNAs were utilised. The spike ins were used to control for efficiencies in the RNA isolation procedure and cDNA synthesis reaction, respectively. Furthermore, prior to the current study we performed several pilot studies where we did not control for plasma hemolyzation. Based on the significant variability in ct values between samples it was very clear that it was crucial to correct for this phenomenon. The fact that erythrocytes contain numerous of miRNAs [44] that unavoidable will contaminate a plasma sample, if the erythrocyte burst during sampling, emphasizes the importance of introducing a hemolysis control step in the study design. To this date, we are not aware of any exercise or training study that takes hemolyzation into consideration when measuring ci-miRNAs. We believe that our combined quality control and normalisation strategies assist with establishing a “best practice” in the field of circulating miRNA measurements and future studies should clearly describe the strategies they take to control for variations in sample collection, extraction, and analysis.

In contrast to the general decrease immediately after exercise, we observed a majority of the significant changes in ci-miRNAs to be increased 1 h and 3 h post exercise. Interestingly, three muscle-specific ci-miRNAs (miR-1, miR-133a and miR-133b) were either significant or borderline significantly up-regulated 3 h post exercise. This finding is in line with our previous finding showing that miR-1 and 133a are up-regulated in skeletal muscle after an acute exercise bout [37], suggesting secretion from muscle or muscle damage. Furthermore, the liver enriched miR-223 was up regulated 1 h after exercise. MiR-223 is used as a biomarker for patients with hepatocellular carcinoma and chronic hepatitis [45]. To our knowledge, no studies have described miR-223 regulation in any tissue in response to acute exercise or endurance training. Other liver enriched ci-miRNAs, such as members of the miR-143/145 were increased after exercise in the current study. MiR-143 and miR-145 are up regulated in the liver of obese mice and miR-143 negatively regulates the insulin signalling pathway [39]. While extreme endurance exercise can cause acute liver damage the exercise protocol used in the current study should not, and is not likely responsible for the increase in liver enriched ci-miRNAs. Taken together, the liver seems to be highly affected by exercise and endurance training in terms of ci-miRNA secretion and should be further investigated in future exercise studies (Table 2).

In the current study, we observed that the miR-103/107 homologs were both increased at rest after 12 weeks of training. Mir-103 and miR-107 are co-regulated in several studies [46], [47] and are major regulators of glucose homeostasis in the liver and adipose tissue [47]. In addition, the muscle specific miR-133a was down regulated at rest after 12 weeks endurance training. We and others have previously shown ∼50% decreases in miR-133a expression in human skeletal muscle in response to 12 weeks of endurance training [10], [37]. Similarly, a decrease in the circulating miR-92a at rest in response to training is consistent with the training induced expression pattern previously observed in skeletal muscle [10]. However, where the observed decrease in ci-miR-29b expression in the current study is in line with the expression pattern in skeletal muscle previously described [10] the expression pattern is the opposite of recent findings by Russell and co-workers [12]. MiR-29b has been linked to myotube differentiation and could therefore potentially play a role in the regulation of muscle adaptation to endurance training [48], [49].

Importantly, several cardiac and muscle specific ci-miRNAs were not detectable in the circulation neither at basal nor after acute exercise. For instance, miR-206 that is clustered in the genome with miR-133b and specifically expressed in skeletal muscle could not be detected. In addition, the cardiac specific miR-208 was not detectable. Together this suggests that the increases in muscle specific miRNAs observed in the current study is due to selective secretion rather than generalised passive release due to muscle damage or muscle biopsies. The observation that some ci-miRNAs are not secreted despite high intracellular expression levels are in line with the observation that cellular miRNA secretion mechanism is a selective process where some miRNAs are destined to be secreted and others remain intracellular [50].

A large percentage of our sample set was above the hemolysis threshold even though the samples were not visually hemolyzed. Due to low sample quality we were obliged to exclude 50% of the samples in the first quality control. The only major difference from plasma RNA isolation recommendations [33] was that we centrifuged our samples at 4°C and not at room temperature. In the second quality control step we excluded three additional samples sets because the erythrocyte specific miR-451 was too highly expressed and we therefore assumed sample hemolysis. However, miR-451 is also highly expressed in skeletal muscle and has been shown to be increased in response to resistance training in some individuals [11]. Thus, we might have missed a differentially expressed exercise induced ci-miRNA based on that assumption. In addition, we could also have missed a few potential important miRNAs in the screening procedure. By using the screening approach where we pool cDNA from samples for each time-point we do not take abnormal response patterns from individual into consideration.

In conclusion, endurance exercise and training induce changes in the ci-miRNA plasma signature. These changes are dynamic over shorts periods of time in acute exercise as well as over longer period time of endurance exercise training. Importantly, muscle specific ci-miRNAs were regulated consistently with the regulations observed within the skeletal muscle in response to exercise [37]. Future studies are necessary to determine whether these altered ci-miRNAs have a physiological role by which endurance exercise affects whole-body metabolism.
